# Self-Organization and Genomic Causality in Models of Morphogenesis

**DOI:** 10.3390/e25060873

**Published:** 2023-05-30

**Authors:** Ute Deichmann

**Affiliations:** The Jacques Loeb Centre for the History and Philosophy of the Life Sciences, Ben-Gurion University of the Negev, Beer Sheva 84105, Israel; uted@post.bgu.ac.il

**Keywords:** reaction–diffusion models in morphogenesis, pattern formation, developmental gene regulatory networks, Alan Turing, Eric Davidson

## Abstract

The debate about what causes the generation of form and structure in embryological development goes back to antiquity. Most recently, it has focused on the divergent views as to whether the generation of patterns and form in development is a largely self-organized process or is mainly determined by the genome, in particular, complex developmental gene regulatory processes. This paper presents and analyzes pertinent models of pattern formation and form generation in a developing organism in the past and the present, with a special emphasis on Alan Turing’s 1952 reaction–diffusion model. I first draw attention to the fact that Turing’s paper remained, at first, without a noticeable impact on the community of biologists because purely physical–chemical models were unable to explain embryological development and often also simple repetitive patterns. I then show that from the year 2000 and onwards, Turing’s 1952 paper was increasingly cited also by biologists. The model was updated to include gene products and now seemed able to account for the generation of biological patterns, though discrepancies between models and biological reality remained. I then point out Eric Davidson’s successful theory of early embryogenesis based on gene-regulatory network analysis and its mathematical modeling that not only was able to provide a mechanistic and causal explanation for gene regulatory events controlling developmental cell fate specification but, unlike reaction–diffusion models, also addressed the effects of evolution and organisms’ longstanding developmental and species stability. The paper concludes with an outlook on further developments of the gene regulatory network model.

## 1. Introduction

Self-organization as the spontaneous emergence of spatio-temporal patterns through physical or chemical processes has been described in many different systems, for example, in non-living reaction–diffusion systems, such as the Belousov–Zhabotinsky reaction. It was used for an explanation of morphogenesis by Alan Turing in 1952 [1] More recently, it came to prominence in embryology with the use of stem cells and their in vitro differentiation into various tissues, and self-organization has become a fashionable topic in studies of the development of patterns and form.

The idea of self-organization—in various forms and terms—has a long history, and the question of the generation of shapes and structures in embryological development, in general, has occupied and fascinated philosophers and scientists for centuries, inducing them to adopt opposing views: the belief that the structures of adults were existent in miniature in the germ cells or programmed in the genes or genome, contrasted with the conviction that new forms and structures were newly created in the embryo.

This debate about what causes form and structure formation in the growing embryo goes back to antiquity. On the one hand, there was the idea of material continuity between generations that were expressed, for example, in the theories of pangenesis, according to which all organs of the body of a parent produce invisible “seeds” that were transmitted during sexual intercourse, or in the theory of preformation, according to which the structure of an adult organism was already preformed in the germ cells. On the other hand, development was understood as a process of increasing complexity from an unorganized egg that was brought about either by immaterial forces or by self-organizing matter. The former view originated in the School of Hippocrates, while the most prominent protagonist of the latter one (that in the 17th century was termed epigenesis) was Aristotle.

With the advent of experimental biology and particularly the enormous progress in cytology in the late 19th century, a new debate arose about self-organization in development. In the early 20th century, cytologists, such as Theodor Boveri in Germany and Edmund Wilson in the United States, provided ample experimental evidence for the central role of the cell nucleus, chromosomes, and genes in development [2]. However, the notion of a prominent role of the nucleus in development was strongly opposed by experimental embryologists, in particular the influential school of Hans Spemann in Germany. According to Spemann, the cytoplasm was the causal agent of development, not the nucleus; developmental steps were connected by a complicated web and determined by cytoplasmic factors as a kind of self-organized process.

In the 21st century, the debate continued between protagonists of the notion that regulation by genomic genes is the primary cause for the generation of form in embryonic development and those who believed that development is largely self-organized and that it is not genetically determined or regulated. The most prominent representative of the former view was Eric Davidson, who believed that the analysis of complex, hierarchical, multigene developmental gene regulatory networks offers an understanding of the precise spatial and temporal pattern of gene expression of an entire developmental process [3,4]. The latter view is held by embryologists and computational biologists who use modified reaction–diffusion models to simulate pattern formation in embryogenesis. An example is the group of Patrick Müller, according to which “embryonic development is a largely self-organizing process” and who have extended the reaction–diffusion theory to “realistic multi-component networks” [5].

In this paper, I present and examine (1) pertinent physical–chemical and genome-based models of pattern formation and morphogenesis in the past and present, with a special emphasis on Alan Turing’s reaction–diffusion model and its reception in the community of biologists, and (2) recent attempts to combine physical–chemical models with models of gene regulation. By showing the insufficiency of purely physical–chemical models for the explanation of embryological development and often also of organisms’ repetitive patterns, I claim the relevance to models of development of Brenner’s dictum that “Biological systems are information-processing machines, and this must be an essential part of any theory we may construct” [6]. I point out Eric Davidson’s successful model of early embryogenesis based on gene-regulatory network analysis and its further development by Ellen Rothenberg and James Briscoe, who also address some of the model’s shortcomings, such as a lack of consideration of tissue mechanics and quantitation.

## 2. Prominent Models of Self-Organization in Morphogenesis and Their Critics

### 2.1. D’Arcy Thompson: Mathematical Modeling of Organisms’ Growth and Form

While Mendel’s mathematical modeling of hybridization in plants was one of the earliest and most fruitful models in the study of heredity and biology in general, British zoologist Wentworth D’Arcy Thompson was one of the early theoreticians of self-organization based on the laws of mathematics and physics. His major work, *On Growth and Form* (1942) [7], has often been commented on, and I review his major theses here only briefly because of their influence on Alan Turing. Like Mendel, Thompson perceived mathematics not only as a tool for representation and explanation but as an expression of biological reality. According to him, “the mathematical definition of a ‘form’ has a quality of precision which was quite lacking in our earlier stage of mere description”; this brings us “in touch with Galileo’s aphorism that ‘the Book of Nature is written in characters of Geometry’” [7]. Similarly, Plato’s primacy of form over matter and Kant’s dictum that the criterion of true science lay in its relation to mathematics played a major role in Thompson’s reasoning.

In his widely read book, *On Growth and Form*, first published in 1917 [7], Thompson combined morphology with simple mathematics and Greek philosophy to find unifying principles in life’s forms. According to him, the organic form was a diagram of forces predetermined by the physical organization of the system in which it developed. His “theory of transformations” aimed at showing how the differences between forms of related species, in particular fish, could be represented geometrically so that one form could be transformed into another one with the help of a simple equation. As an anti-materialist, he rejected theories that attributed specific properties to particles of the protoplasm, such as chromosomes. In his opinion, such an attribution would mean committing the “error of attributing to matter what is due to energy and is manifested in force: or more strictly speaking, of attributing to material particles individually what is due to the energy of their collocation.” To him, August Weismann’s term of a “hereditary substance” could only mean “that that particular portion of matter is the essential vehicle of a particular charge or distribution of energy, in which is involved the capability of producing motion, or of doing work” [7] (p. 288). Thompson also rejected Darwin’s idea of gradual evolution through natural selection because, according to the Platonic idea of pure form (idea), mathematical shapes cannot be transformed through gradations, and organic forms are fashioned by the direct action of physical force, not by selection.

Thompson emphasized the importance of osmotic models of morphogenesis, for example, the work by physical chemist Stéphane Leduc, who claimed to have created artificial life by simulating phenomena such as karyokinesis and organisms’ forms with the help of osmotic growth processes (ibid., pp. 324, 501). Leduc did not search for a causal explanation of these phenomena, and he called into question the validity of the generally accepted cell theory of Remak and Virchow of the 1850s, according to which cells arise only through the division of existing cells [8].

Thompson’s view contradicted the convictions of prominent biologists at the time who had begun to examine the specificity of basic life processes and organisms’ ability to regulate them. Examples are Jacques Loeb, according to whom the artificial creation of life was not only a physical process but had to involve the synthesis of specific molecules, in particular, self-replicating DNA (at the time referred to as nuclein) [9], and Hans Driesch, who held that these osmotic patterns and shapes lack the reproducible specificity of organic forms and the capacity to self-regulate [10].

Thompson’s book has been widely admired and praised by a number of renowned scientists, but it had little direct scientific impact on research and never contributed to mainstream experimental biology at any time. However, there is a recently renewed appreciation for the mathematical and physical approaches of Thompson and his predecessors, such as Wilhelm His: morphologists have begun to combine the old, largely metaphoric approach of Thompson and others with insights from molecular biology, such as gene regulation and signaling molecules [11]. Molecular embryologists James Briscoe and Anna Kicheva [12] believe that Thompson’s notion “that physical laws constrain biological systems has far reaching consequences”. Thompson’s book inspired many mathematicians and theoretical biologists to mathematically simulate pattern formation, and it pointed to the insufficiency of neo-Darwinian evolutionary theory, inspiring Stephen J. Gould’s criticism of gradualism and adaptationism [13,14]. However, Briscoe’s and Kicheva’s reminder that mathematical constructions “do not in themselves provide a causal explanation for biological form. This requires molecular, genetic, or mechanical insight into the processes”, is relevant not only to D’Arcy Thompson’s models but also to all subsequent models of morphogenesis.

### 2.2. Alan Turing’s Mathematical-Chemical Model of Self-Organization in Morphogenesis and Its Reception

The paper “The Chemical Basis of Morphogenesis” by mathematician and computer scientist Alan Turing [1] has played a central role in the discussion about self-organization in morphogenesis. This paper has been more frequently cited than the rest of his work taken together [15], though, interestingly, a citation analysis in the Web of Knowledge shows that a noteworthy increase in the number of citations per year only occurred in the early 2000s (see Section 3). It has recently inspired embryologists and computational biologists to generate models of pattern formation in development. As Peter Saunders [15] has pointed out, the title of the paper and the term “morphogen”—form producer—are not quite correct because the paper mostly dealt with the formation of patterns, not form.

In the introduction to his paper, Turing [1] suggested “that a system of chemical substances, called morphogens, reacting together and diffusing through a tissue, is adequate to account for the main phenomena of morphogenesis”. His emphasis that “the theory does not make any new hypotheses; it merely suggests that certain well-known physical laws are sufficient to account for many of the facts”, indicates the influence Turing received from D’Arcy Thompson.

Turing aimed to demonstrate that patterns can be created spontaneously in an originally homogeneous cell. To explain how this can happen, that is, how spatial patterns in an egg can form autonomously, he introduced reaction–diffusion equations into the modeling of development. He succeeded in showing mathematically that in a system of two or more diffusing reagents, a pattern of high and low concentrations can spontaneously emerge from an initially uniform distribution [1]. The idea was that two homogeneously distributed substances within a certain space, one “locally activated” and the other capable of “long-range inhibition”, can produce novel shapes and gradients. The results of these substance interactions are dependent on just four variables per substance—the rate of production, the rate of degradation, the rate of diffusion, and the strength of their activating/inhibiting interactions.

Turing began to work on morphogenesis in the context of his work on the design of thinking machines, which raised his curiosity about the design of brain development. According to his biographer Alan Hodges [16] (pp. 541–542, cited in [17] p. 89), “There were two possibilities: either a brain learnt to think by dint of interaction with the world, or else it had something written in it at birth—which must be programmed, in a looser sense, by the genes. Brains were too complicated to consider at first. But how did anything know how to grow? There lay the question”. Turing became fascinated with embryology, the taking shape of an animal from the sphere of a cell, and the fact that, as he believed, nobody had thought about what determined this growth (ibid.).

Another reason for Turing to become interested in biology was his desire to “defeat the argument from design” as proof of the existence of God. This argument was still widespread, although Darwin’s materialistic theory of evolution was widely accepted at the time [15]. Therefore, Turing followed Thompson, who had urged biologists to attempt to explain forms in the same way physicists do, namely by reference to mechanical forces (ibid.), though, unlike Thompson, Turing focused on the generation of patterns, not of forms. It is surprising—and has been noticed by many commentators—that Turing approached the task he set himself mostly on his own, without consulting colleagues from biology or taking notice of what other modelers in biology did. There are only very few references in his paper; they include Thompson’s *On Growth and Form*. According to Saunders [15], this reflected Turing’s way of working, i.e., to determine what was important and not to be diverted from his view by what others did.

This attitude may, in part, explain the contradictions in the paper’s premises and the grave shortcomings regarding the state of the art in biological research. Turing’s “mathematical model of the growing embryo” was indeed, as intended, simple and elegant. However, to consider the embryo as a state function and eliminate growth is in direct contradiction to his stated goal. While diffusion and osmotic pressures are widely dealt with, the “chemical reactions” are not related to any particular type of molecules or their specificities, though at the time, biological specificity was largely related to proteins. Most importantly, the concepts of gene and cell were unclear, and the “genes as enzymes” theory that Turing advocated was long obsolete. In Turing’s words: the genes may “be considered to be morphogens”; “it would be more accurate (...) to regard them as radicals of the giant molecules known as chromosomes; … the function of genes is presumed to be purely catalytic. They catalyze the production of other morphogens, which in turn may only be catalysts” [1].

The gene-as-enzyme hypothesis that was proposed by Richard Goldschmidt in 1927 soon proved to be untenable for various reasons [18]. Since the 1930s, several developmental geneticists have studied the cooperation of genes and their biochemical effects. In 1941, based on X-ray studies of mutants in the mold Neurospora, the American geneticists George Beadle and Edward Tatum found that each gene governed the production of one specific enzyme—the “one gene-one enzyme” concept. This means that the characteristic function of the gene was to supervise the formation of a particular enzyme. The authors determined that all biochemical reactions in an organism were controlled by specific genes, work for which they shared, with Joshua Lederberg, the 1958 Nobel Prize in Physiology and Medicine. Apart from overlooking this important advance in the chemistry of development, Turing disregarded the fact that the assumption of enzymes catalyzing the production of other enzymes (catalysts), etc., would lead to an infinite regress, an observation which, a few years later, led Francis Crick to conclude that the synthesis of enzymes must be radically different from the synthesis of other molecules and that the existence of a template seemed the only logical solution to this dilemma [19].

This disregard for biological knowledge and logic supports Evelyn Fox Keller’s assessment that Turing was more interested in “mathematical fruitfulness and accessibility” than in the correspondence of his hypothetical reactions to real reactions in the cell [17]. Biologists, on the other hand, were not interested in whether the interactions could build patterns the way Turing suggested but whether they really do. Additionally, for a long period of time, there was no evidence of it. For this reason, the model was hardly cited by biologists for decades (a detailed historical analysis of Turing’s model is in [17]. One of the few biologists interested in the model was Conrad Waddington, but he, too, in a letter to Turing in 1952, raised “several concerns about the applicability of Turing’s reaction-diffusion model to biological developmental systems, questioning its limitation to reproduce some observed behaviors in embryonic development such as pattern scaling with tissue size or the generation of a spatial pattern of discrete cell types” [20]. Waddington believed that the model might apply to the formation of patterns such as spots and stripes but not to morphogenesis.

In the 1970s, Ilya Prigogine and his school of the irreversible thermodynamics of complex systems made the model popular for some time. The number of reaction–diffusion studies increased, particularly pattern formation in butterfly wings and animal coats. Scientists applied updated versions of Turing’s model and other mathematical models to simulate pattern formation in a variety of different animal systems, such as the generation of periodic seashell patterns and body segmentation in Drosophila. These early studies of pattern formation, for example, the work by Hans Meinhardt and Alfred Gierer, have been described and analyzed in detail by Siegfried Roth [10].

However, for a variety of reasons, many of these simulation models did not reflect reality. One of the reasons is that, like Turing, their authors disregarded genes not only as causal factors for morphogenesis and development as a whole but also for many biochemical pathways for pattern formation. They disregarded the fact that the unfertilized egg, as was shown by Christiane Nüsslein-Volhard, was not a homogeneous sphere but rather a highly organized structure containing, among other things, a spatial pattern of information carrying mRNA and proteins [17] (p. 111). Additionally, many of these models also disregarded the difference between pattern formation and the complex processes of embryogenesis, a problem that, as Francis Crick remembered, may have been perceived even by Turing himself: At a meeting on mathematical models of development in 1972, Crick, one of the skeptics regarding the validity of Turing’s model for development, quoted Turing’s remark about the zebra: “Well, the stripes are easy but what about the horse part?” [21]. Pattern formation can be modeled elegantly and relatively simply, but morphogenesis and development would require modeling of the zebra itself, its body architecture, organs, etc., in a very complex way that would also have to take into consideration developmental constancy and evolution.

Evolutionary developmental biologist Michael Akam, who has studied the generation of the repeating stripes along the antero–posterior axis of Drosophila for many years, in 1989, wrote a widely discussed paper with the title “*Making Stripes Inelegantly*” [22], in which he discussed two possible ways of generating the exact periodicity of the stripes: An “elegant mechanism” that was favored by model builders such as Meinhardt [23] or Lacalli et al. [24], and that would “use an intrinsically periodic pattern-generating system, comprising the pair-rule genes [a class of segmentation genes] and their products.” It only needed to be triggered by local stimuli from the gap genes that control the early cascade of the segmentation pathway. The alternative was that “unique instructions could be generated by the gap-gene proteins to define the position of each pair-rule stripe”. His analysis of the interaction between the different kinds of genes (gap and pair-rule genes) showed that the less elegant “specific instruction” process was more likely to take place in the organism and that “the apparent simplicity of the repeating segment pattern” was deceptive. He concluded that spontaneous pattern-generating mechanisms might contribute to “sharpen the boundaries between stripes” but that they do not define periodicity.

**Figure 1:**
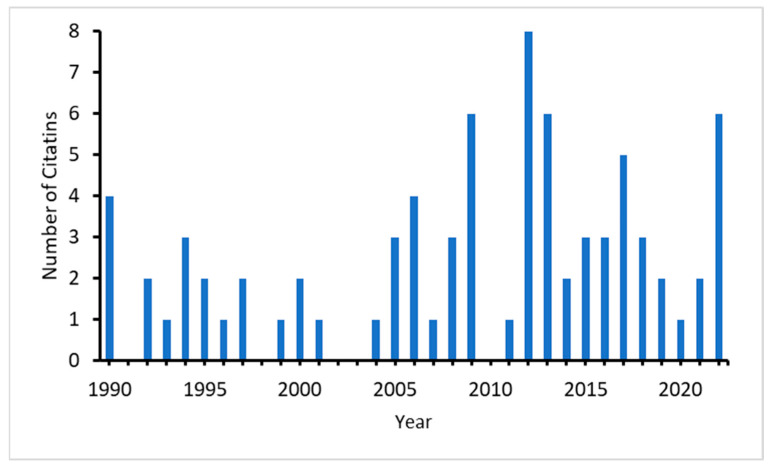
Number of citations of Akam (1989) in the Web of Science.

Akam’s paper [22] has been continuously cited since its publication, with a significantly higher average number of citations since 2005 (Figure 1). Most of the citing papers appeared in journals of developmental biology and computational biology and, more recently, also in physical and mathematical journals.

Among the citing authors are J. Sharpe and A. Economou (whose work is briefly presented in Section 3). More than 30 years later, Akam and his collaborators proposed a new mechanism for the segmentation of Drosophila and other arthropods, in which conserved gene regulatory networks play decisive roles, concluding that “over the past four decades, arthropod segmentation has contributed enormously to our understanding of developmental gene networks and their evolution” [25].

The strongest critic of mathematical simulation models that are not based on experimental perturbation was Eric Davidson. According to him, “one of the worst fallacies [in the field of modeling in biology] is the assumption that if you can make a model, which simulates a process, then the model must represent how it works. The great example is Meinhardt’s explanation of Drosophila stripes in terms of reaction-diffusion equations. He explained it perfectly, except it doesn’t happen to be how it works. [...] And what showed us how it works, of course, was taking the DNA out and experimentally finding out how it works” [26]. Davidson’s successful attempt to causally explain the molecular events of early development in sea urchins with the help of his theory of gene regulatory networks and to generate a Boolean model for it is briefly discussed in Section 4.

## 3. The Recent Revival of Turing’s Theory of Morphogenesis and Other Theories of Self-Organization in Biology; Merging with Genomic Models

Taken alone, methods based on Turing’s model and updated reaction–diffusion models by others so far have been unable to explain the complex developmental program that is brought about by multiple genetic and molecular pathways; they even cannot account for many of the simpler patterns, such as stripes. However, according to Marcon and Sharpe [27], Turing-type reaction–diffusion models and other models of self-organization have recently started to be taken more seriously and applied to a variety of patterning processes by biologists and not only by mathematicians.

**Figure 2:**
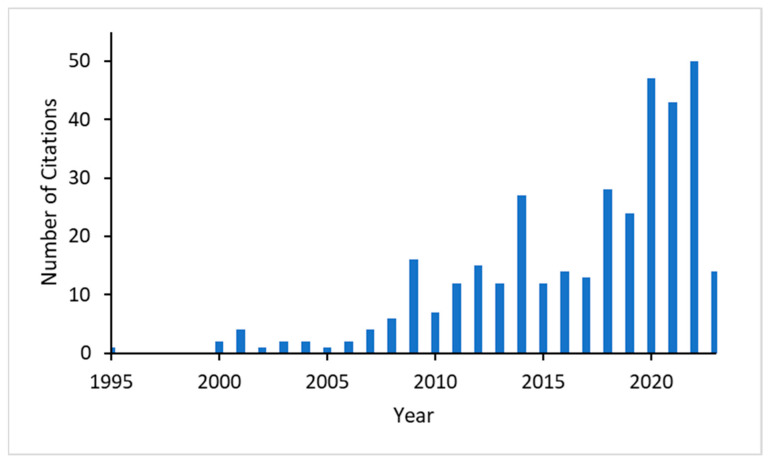
Number of citations of Turing’s “The Chemical Basis of Morphogenesis” [1] in the Web of Knowledge, starting in 1995.

Citation analysis in the Web of Science shows that the number of citations of Turing’s paper has drastically increased after 2000, especially since 2020 (Figure 2). Of the 214 citing papers since 2020, ca. 62 covered biological topics. Thirty-four of them dealt with topics of pattern formation and morphogenesis, several of them including gene regulatory networks in the title, and twenty-eight papers dealt with other biological topics, such as ecology, evolution, and neural networks.

Multi-component Turing networks that do not require differential diffusivity have been proposed by Patrick Müller and his group; the authors believe that embryonic development is largely a self-organizing process [5]. Most of their simulation models have not been experimentally tested.

Self-organization has also been argued to be responsible for symmetry breaking (i.e., the acquisition of asymmetry along an axis) in the early mammalian embryo [28]. The authors hold that symmetry can be broken through stochastic variations in the cytoskeleton structure, but they perceive a difference between experiments conducted in vivo and in vitro. In in vivo studies, it is maternal and/or extraembryonic tissues that are instrumental in the establishment of an anterior–posterior axis through asymmetric signaling activity, whereas, in studies using in vitro cultures of blastocysts or stem cell aggregates, this does not seem to be necessary for symmetry breaking. According to Stas Shvartsman [29], genomic controls guide the self-organizing processes, selecting specific outcomes from many different possibilities.

Eric Karsenti [30] believes that the whole cell cycle in eukaryotes “can be seen as being based on the principle of self-organization by reaction-diffusion, both temporally and spatially. But he made it clear that though microtubule patterns appear self-organized, mutated cells (in the Ser-Thr protein kinase Orb6) have a different shape and microtubules cannot organize in long bundles. This means that genes are required for the self-organizing process. He also thinks it important to realize that none of these processes are true Turing patterns”, because “the symmetry is not broken by spontaneous instabilities, but rather by deterministic effects”, such as cyclin synthesis for the oscillator and stereospecific targeting of a small G-protein exchange factor to chromatin for nuclear and spindle assembly.

Tom Misteli [31] views the genome as a self-organizing system because this perception makes it possible to understand the conflicting aspects of genome organization, namely the stability of the transcriptional program of a given cell on the one hand and the dynamic and stochastic nature of gene expression on the other. According to Misteli, the available data support the notion that the major features of higher-order genome architecture are emergent properties in a self-organizing system that is driven by the functional status of the genome. He defines self-organization as “the inherent tendency for systems to form coherent patterns solely based on the dynamic interaction of its components,” in the case of the genome, “the physical interactions of proteins with chromatin and of chromatin with chromatin” [32].

Misteli is of the opinion that “the architectural properties of the genome are driven by the sum of activities [such as gene activity] that occur along the genome”, most of which are affected by DNA sequence. According to him, “the DNA sequence is a major contributor to determining these activities”, although “most DNA-binding proteins bind far more promiscuously than we have previously thought”. He believes that chromatin remodeling complexes promiscuously bind to chromatin and remodel it. The DNA sequence-specific proteins are important during the short period of time in which chromatin is open. All of this suggests that the term self-organization in Misteli’s definition only applies to the genome after the activities along the genome have already been established, most of them by DNA sequence-specific events. From this, it can be concluded that: the genome is self-organized this self-organization is mainly based on previous DNA sequence-specific events.

Perspectives on the promiscuity of DNA binding proteins and remodeling events differ among different researchers. According to James Briscoe [33], promiscuity in the binding of chromatin remodeling complexes exists, but the modifying factors are guided by transcription factors and other regulatory factors that are specific to a DNA sequence. As an example, he mentioned that the starting point of polycomb group regulatory proteins is determined by DNA sequence [34].

Ellen Rothenberg thinks that the difference between sequence-specific transcription factors and chromatin remodeling factors is that the sequence-specific factors require interaction with some more-or-less specific DNA sequence in order to bind, whereas the chromatin remodelers and modifiers do not. She points to the highly complex nature of the binding of the factors: “For each transcription factor, there is still a range of variants of the preferred DNA sequence that are bound with different strengths. So, while all of these sequences (“motifs”) are non-random and statistically far different from background DNA, they are not equivalently good targets for the transcription factor’s binding and can be bound conditionally, for example, better if the chromatin is open than when it is closed” so that the binding is not 100% certain [35] (see also Section 4).

Some recent research has begun to study various cases of pattern formation in animals by combining reaction–diffusion models or other physical–chemical mechanisms with genome-based mechanisms. Examples are Sharpe et al.’s [36] work on the control of digit patterning by a Bmp-Sox9-Wnt Turing network modulated by morphogen gradients and Economou et al.’s [37] perturbation analysis of a Turing-like reaction–diffusion stripe patterning system. Sharpe et al. showed how digit patterning appears to be controlled by a Turing network implemented by gene products. The problem with this study is that though the findings are based on experimental data, some assumptions are speculative, derived from purely mathematical reasoning [38].

The perturbation analysis of a Turing-like reaction–diffusion stripe patterning system—of ridges in the mammalian palate—and the regulatory interactions involved in this process by Economou et al. is another attempt to integrate physical and genomic models. The study shows the cooperation of growth factor ligand proteins, their receptors, genes, and other factors, and it also reveals still existing discrepancies between the results of mathematical modeling and biological reality: the patterning of the palate uses five pathways in the organism, though only two would be required by mathematical modeling. Moreover, to my knowledge, none of these models or other mathematical models have addressed the questions of how models can account for the species specificity of the patterns and their stability in geological time.

Some authors attempt to establish a connection between the metaphor of “epigenetic landscape” that embryologist Conrad Waddington proposed in 1940 and physicists’ notion of an “epigenetic state”, a system-level stable state that arises from the interactions of genes as Waddington had envisioned. They regard Waddington’s vision as a major contribution to the current convergence of molecular and physico-chemical approaches [39,40].

In their review of recent large-scale mathematical analyses of Turing patterns in biology that have attempted to narrow down potential design principles, Sean T. Vittadello et al. [41] showed that despite progress in many areas, the original problems related to the use of Turing models in the context of biology have not yet been fully resolved. One of them was the contradiction between “the beauty of mathematical models” and “the ugly truth of reality”. They discuss an aspect of model development in biology that they consider essential for confronting this problem, namely “the extent to which the assumptions underlying our models are robust and in line with what we see in nature”, describing the “caveats that need to be considered in designing a synthetic Turing-patterning mechanism that is viable in vivo”.

## 4. Models Based on the Concept of Genomic Causality in Development

### 4.1. Eric Davidson’s Model of a Complex Developmental Regulatory Gene Network (GRN)

The most successful model of the description and causal explanation of the early development of a complex organism, the sea urchin, is Eric Davidson’s model of developmental gene regulatory networks (GRNs). Based on decades-long molecular biological research into how cell-type specific gene expression patterns appeared, Davidson adopted a systems approach that included almost all regulatory genes as soon as DNA sequencing was available [42]. However, “experimental perturbation and predictive challenge of the system” remained essential to reveal the underlying causal mechanisms [43].

Davidson created the concept of developmental GRNs in the early 2000s. Basic knowledge of genetic regulation in the development of higher organisms had already been obtained from Drosophila by Christiane Nüsslein-Volhard and Eric Wieschaus, who also demonstrated the hierarchy of maternal genes in the embryo that played an important role in Davidson’s GRN. The early models of the temporal dynamics of already known gene networks in development included only a few genes [17] (pp. 250–251). Davidson was the first to achieve an almost complete model of a regulatory gene network for the development of a particular phenotype (endomesoderm specification) and to construct a mathematical model to account for observation in a complex biological object. Using big sequencing and expression data, he made a large quantitative step from a few regulatory genes to networks of hundreds of genes.

These developmental GRNs contain, as crucial elements, specific cis-regulatory modules (DNA regions binding the transcriptional machinery in the vicinity of the genes they regulate) that direct the expression of developmental transcription factors and signaling molecules. Cis-regulatory modules are causality-inferred regulatory regions of genes that are identified experimentally [44].

A world leader in molecular embryology, Davidson demonstrated that, at least in sea urchins, early development is entirely regulated by the genome. This was, to him, a logical necessity and requirement for evolution because without such a genomic regulatory program, it cannot be ensured that within each species, the outcome of development is extremely reproducible. Davidson’s and his collaborators’ attempts to explain gene regulation in development beyond the study of individual genes started in 1969 [45]. It not only led to the experimental construction of hierarchical GRNs for development and later to their mathematical modeling but also to the proposition that changes in the architecture of GRNs through changes in genomic sequences may be the engine of evolutionary changes in animal body architectures and other major characteristics.

Davidson was one of only a few scholars who, together with Douglas Erwin, not only proposed hypotheses for a causal mechanistic explanation of evolutionary change but also of evolutionary stasis based on the stability of developmental outcomes [46]. The extreme conservation of certain morphological features over immense geological periods gave rise to the question of how these parts of the GRN structure could be stabilized through deep time, questions that found a partial answer in the organizational hierarchy of these structures—the effect of changes differs fundamentally according to where in the network they occur. Small changes continuously occur at the periphery of networks, where effector genes code for proteins, while stasis of network patterning can be found in other parts.

Early on, Davidson joined forces with physicists and computer scientists to integrate computer-generated big data into a systems approach that was based on experiments and aimed at elucidating mechanisms and causal relationships. In 2006, he introduced the term “regulatory genome” for the interactions between regulatory genes and their products during development [3]. This concept was conceived and developed through decades-long, painstaking experimental research by Davidson and his collaborators. They systematically examined the cell-type-specific gene expression patterns before moving on from the “gene-by-gene characterization of the sea urchin embryo to full comprehensiveness” [42]. This systems approach was made possible when sequencing data of the whole sea urchin genome became available. Observations and descriptions were crucial as a starting point. A perturbation analysis was essential because “only by deliberate experimental perturbation and predictive challenge of the system can the mechanisms by which it operates be revealed” [43].

Davidson’s first mathematical models in the 1990s not only contained logic functions (AND, OR, NOT) between the input of different regulatory proteins (transcription factors) but also assumed that they were quantitatively modulated; thus, he and his collaborators created systems of differential equations with continuously variable inputs and outputs [47] (see the overview by Ellen Rothenberg [42]). However, because the key rate constants and concentrations needed to render these models predictive did not exist, Davidson envisaged that a Boolean model, in which the status of each gene is assumed to be either “on” or “off”, might be sufficient as a predictive systems model of development. Together with Isabelle Peter, Davidson converted the whole GRN system into a Boolean model, a “grueling effort lasting many months of concentrated work” [4] (p. 309). This model contained all current data for the logic of transcriptional inputs at each gene (cis-regulatory) system and for the location of each cell at each time point of development [48].

The first results of the Boolean modeling showed that there were only a few inconsistencies between model predictions and measured in vivo gene expression. This meant not only that the key regulatory elements of the GRN and their interactions were almost complete but also that other factors, such as changes in chromatin structure, did not appear to be relevant at this early developmental stage. The model was based on experiments that proposed linkages based on perturbations, not only correlations. It provided a means for experimentally testing the relevance and consistency of the GRN concept, thus fundamentally contrasting with models that merely simulated phenomenological features without analyzing their mechanisms and causes.

Davidson’s GRN model underlines the relevance of three principles that are relevant for the explanation of complex biological systems, namely, informational hierarchy, genomic causality, and biological specificity (see [49]).

### 4.2. Assessments and Further Developments of Davidson’s Developmental GRN Model

Davidson’s developmental GRN model has been successfully used to explain aspects of development in a wide range of different organisms. Like any other model, it has been challenged by new research and is being transformed accordingly. Thus, the original proposal of deep evolutionary conservation of network kernels does not seem to be maintainable—as Douglas Erwin [50] has shown, there is an extensive rewiring of GRN sub-circuits. Despite these changes in the original network concept, the ideas of the central role of GRNs for embryological development and developmental constancy, their hierarchical organization, at least concerning genetic information, and the causal role of genes, have been confirmed in numerous different studies and have remained fruitful.

Ellen Rothenberg emphasized the general importance that Davidson’s and his collaborators’ work on developmental GRNs has had for many different systems. According to her, it is now widely recognized that GRN analysis is a major step to advancing from a descriptive to a mechanistic understanding of biological systems [51], and she showed how much researchers in her own field, hematopoiesis, have benefitted from the pioneering work of Davidson on network control in sea urchins. At the same time, she points to differences between the network models for nonvertebrate embryogenesis and for hematopoietic systems in mammals, particularly regarding dose dependence and timing: in contrast to a rapidly unfolding cascade of transcriptional change guided by transcription factors (TFs) in embryological development, the development of lymphocytes from stem cells in mammals is slow, cell fate choices have a strong stochastic component, and timing is highly variable [52]. Another difference is that though TFs that read regulatory sequences in the genome to initiate changes in the expression of specific genes in development as well as in physiological processes, their actions in the latter are constrained by slow-changing chromatin states and by interactions with other TFs. She uses the development of T lymphocytes to show how binding specificity and dynamics, TF cooperativity, and chromatin state changes impact the regulatory functions of key TFs (ibid). An example is Runx transcription factors. They are always binding specific Runx motifs but choose a different ~10,000 sites out of the possible ~1 million to bind in early T cells than the ones they choose in B cells, stem cells, or even in later T cells [53]. This shows that though this binding is not promiscuous, it is also not predictively deterministic. The action of the Runx factors depends on the different contexts at different DNA sites, such as other regulatory proteins in different pathways [54]. There are also cell type-specific factors that affect the modification of the site choice within the constraints of the sequence motif-specific binding, an area that is currently being explored.

Rothenberg also broadened the view on the role of TFs in developmental processes. They not only bind to regulatory sequences, but certain TFs in the T cell specification network model also play an important role in opening chromatin, displacing nucleosomes, and initiating activating histone modifications [55]. She thus echoes the view that was already brought forward by Gary Felsenfeld [56] that histone modification is preceded by a DNA sequence-specific event. Closely examining the collaboration of TFs in their system, Shin and Rothenberg [57] show how “transcription factors collaborate to initiate, stabilize, synergize, oppose, or silence gene expression programs”.

James Briscoe considers the theory of GRN by Davidson and colleagues to be a logical and formal framework in which to describe the transcriptional programs that have to be activated at the right time and place during development, programs that are encoded in the genome. Because the functional output of a developmental GRN is the “organized expression of genes”, the “analysis of the architecture and dynamics of these networks offers an understanding and a rationale for the precise spatial and temporal pattern of expression of the thousands of genes necessary for tissue patterning” [33]. Referring to the example of Davidson’s “rigorous and comprehensive dissection of sea urchin endomesoderm development”, he concluded that this work illustrates the “potential of the GRN approach to provide a mechanistic and causal explanation to a complex set of gene regulatory events controlling development cell fate specification” (ibid.). He also points to the importance of GRNs that have been reconstructed from other species and tissues.

Briscoe also demonstrated some limitations of the GRN approach, in particular, its lack of quantitation and its emphasis on structure and topology, that is, connections between genes and transcription factors. According to him, this “underplays the dynamics and quantitative aspects of a system, which is crucial when feedback and nonlinearity are involved” [33]. Briscoe made it clear that Davidson, in his last years, became increasingly interested in getting these quantitative data, and he is of the opinion that advances in experimental techniques now make it possible to collect and analyze more high-resolution and quantitative data [34]. Briscoe believes that the combination of GRN analysis and dynamic systems approaches also serves to overcome some of the limitations of the GRN approach. According to him, dynamic systems and complexity theory help explain and predict behavior that is not easy to understand otherwise, such as sudden changes in behavior in a deterministic dynamical system, known as bifurcation. Briscoe uses a framework based on Catastrophe theory in order to generate quantitative geometric models of cell differentiation [58].Dynamic systems theory is used as a framework to describe specific developmental mechanisms, define simplifying abstractions, and explain principles. Thus, the interactions within the GRN serve as an example of multilevel behavior that explains how tissue patterns of gene expression arise from the molecular interactions of transcription factors in individual cells [33]. However, many questions still remain open, such as how stochastic fluctuations that are inherent to gene regulation affect performance and are propagated through a gene regulatory network or the role of cell and tissue mechanics in pattern formation.

## 5. Conclusions

Until now, updated Turing models by themselves do not appear to be able to explain either robust morphogenesis or pattern generation in development. A combination of theoretical and experimental approaches and the integration of gene products into models of self-organization or other mathematical models appears most promising for a causal explanation of morphogenesis and pattern formation in organisms. In many cases, however, empirical confirmation is still missing, and the old problem of the discrepancy between model prediction and biological reality has not been solved in most cases. The models usually disregard the effect of evolution and do not address longstanding developmental and species stability. The GRN approach based on a complex set of gene regulatory events has proved most successful in controlling developmental cell fate specification and in providing an explanation for developmental constancy and evolutionary change and stability. However, it also has some limitations, such as its focus on gene regulation and disregard of cell tissue mechanics in morphogenesis [33].

The metaphor of “genetic program” as a molecular genetic concept was introduced by Francois Jacob and Jacques Monod in the context of their work on gene regulation in bacteria [59]. Despite the fact that the analogy to a computer program, which this term may suggest, has been strongly criticized, in particular by historians and philosophers of science, the metaphor has remained influential in biology as a succession of steps, not the equivalent of a computer program [60]. Some years later, Jacob [61] used the metaphor to suggest a modern vision of development that combines the ancient idea of preformation, which for him was the genetic program, and epigenesis that he understood, for example, as feedback regulation of enzyme activities. We may extend this view of epigenesis by including other events that are not directly controlled by the genome, such as the mechanics of cells, adhesion processes between molecules, and geometric constraints of development.

In 2016, Eric Davidson took a similar philosophical stand when he distinguished between two types of experimentally supported causal explanations in his field, animal developmental biology and also in the evolutionary biology of the animal body plan. He described them as “rooted” and “unrooted” explanations. In his words, “rooted causal explanation provides logical links to and from the genomic regulatory code, extending right into the genomic sequences that control regulatory gene expression”; “Unrooted explanations” are those “in which the only causality is to be located within a process considered, for example within a synthesis pathway (without reference to why the enzymes are expressed where they are in the first place), or within a signaling event (without reference to why the signal is expressed in the sending cells, or what it does to gene activation in the receiving cells)” [43]. The many cases of the spontaneous emergence of patterns or forms in biology that seem to be driven by physical or chemical forces and that are therefore labeled self-organized are good candidates for other examples of Jacob’s “epigenesis” and Davidson’s “unrooted explanations”. The concept of self-organization in development as modern epigenesis seems to be most fruitful when it is included in the frame of genomic causality, and models of genomic causality have to integrate physical–chemical models to be complete.
